# Comments on “Antiapoptotic and antioxidant capacity of phytochemicals from Roselle (*Hibiscus sabdariffa)*and their potential effects on monosodium glutamate-induced testicular damage in rat”

**DOI:** 10.1007/s11356-021-16609-0

**Published:** 2021-09-28

**Authors:** Huichia Chao, Masanori Komura

**Affiliations:** International Glutamate Technical Committee (IGTC), Avenue de Tervuren 188A, 1150 Brussels, Belgium

We are writing this letter to comment on study conducted by Gad FAM et al., titled “Antiapoptotic and antioxidant capacity of phytochemicals from Roselle (*Hibiscus sabdariffa*) and their potential effects on monosodium glutamate-induced testicular damage in rat” reporting that *Hibiscus sabdariffa* (HS) beverage can ameliorate MSG-induced testicular toxicity.

This study reported that rats administered MSG (60 mg/kg) by gavage showed a significant decrease in testosterone, luteinizing hormone (LH), and follicle-stimulating hormone (FSH) (see Fig. 1). However, it was also reported that inclusion of MSG (5.2 g/kg bw/day in average) in the diet does not significantly affect the circadian variation of serum levels of LH and testosterone in adult male rats, and only subcutaneous injection of MSG (1g/kg bw) lowered levels of serum LH and testosterone and did so only temporarily (Yonetani and Matsuzawa 1978; Takasaki et al. 1979). Takasaki et al. also reported that serum levels of LH and FSH in adult male rats given large amounts of MSG during weaning did not differ from the levels seen in the control rats. It is known that the effect of MSG administration depends on the severity of hypothalamic damage, and only injection or force-feeding of large amounts of MSG induces hypothalamic damage in neonates (Rascher 1981; Torii et al. 1981; Mestres and Rascher 1983; Seress et al. 1984). Therefore, it is very unlikely that oral administration of such a low dose (60 mg/kg bw) of MSG affects these endocrine hormones. Regarding the dose of MSG, the author cited a study by Hamza RZ, AL-Harbi MS (Hamza and Al-Harbi 2014). However, according to the experimental protocols of this study, they used 6, 17.5, and 60 g/kg bw, not 60 mg/kg; therefore, the author should confirm the actual dose of MSG.

In a human study, Carlson et al. investigated hormone responses to orally administered glutamic acid in 13 subjects at a dose of 10 g of glutamic acid (approximately 150 mg/kg BW). No changes in serum LH and TSH (thyroid-stimulating hormone) were reported even with an approximately 10-fold increase in plasma glutamate concentrations (Carlson et al. 1989). These results were subsequently confirmed by Fernstrom in a study where 8 adult males in a fasting state received the same oral dose (as glutamic acid) of MSG (12.7 g) (Fernstrom 2000). The findings of these studies indicate that acute or short-term oral intake of MSG in humans does not affect pituitary function even when the plasma glutamate concentration is very high. On the other hand, MSG is a food additive commonly used as a flavor enhancer; therefore, it is very unlikely that someone would take a large oral dose of MSG alone. In long-term use, it is well established that glutamate with food is metabolized and mainly serves as an energy source for enterocytes (Wu 1998; Reeds et al. 2000), and normal dietary glutamate intake has only a small effect on blood glutamate concentration in humans (Ghezzi et al. 1985; Stegink et al. 1985).Taking these factors together, MSG intake under normal levels of human consumption does not impact plasma Glu concentration and does not subsequently influence pituitary function, either.

Oral administration with MSG has resulted in decreased glutathione (GSH) in testicular tissue homogenates in the rat (see Fig. 2). However, glutamate, along with cystine and glycine, is known to be a precursor of GSH, an antioxidant which can prevent oxidative damage caused by reactive oxygen species (Wu et al. 2004). In fed piglets, a study using enteral amino acid tracers found that enteral glutamate is the preferential source for mucosal glutathione synthesis rather than the intracellularly generated glutamate (Reeds et al. 1997). Another study in piglets found that dietary supplementation with glutamate alleviated diquat-induced oxidative stress by enhancing antioxidant systems of serum SOD, T-AOC, and NO levels and inhibiting MDA generation (Yin et al. 2015). In a mouse study, a 1.3-fold increase in plasma glutamic acid levels was reported to show a significant increase in the total GSH level in the liver (Kurihara et al. 2007). In a human study using macrophages derived from monocytes, glutamate added to a culture medium in the presence of cystine was reported to increase GSH concentration in a dose-dependent manner (Rimaniol et al. 2001). Recently, the effectiveness of GSH precursors on GSH synthesis in humans was investigated in a 3-month intervention test, and it was found that supplementation of oral GSH precursors (containing all three precursors of GSH: cystine, glutamate, and glycine) increased serum GSH levels across the test groups (Dolbashid et al. 2018). Notably, in contrast to the findings of Gad FAM et al., these results suggest that elevated plasma Glu levels may enhance GSH production in the liver, monocytes, or other tissues.

Finally, the authors found that forced oral administration of MSG at a dose of 60 mg/kg over 6 weeks induced testicular toxicity with histological damage in rats (Figs. 3, 4, and 5); however, Owen et al. has reported that after feeding a diet containing up to 4% MSG (the dose is equal to 4000 mg/kg/day with a food intake of 25 g and body weight of 250g) to rats, there were no histological findings after 12 and 104 weeks (Owen et al. 1978a). These results were also confirmed in a similar study in beagle dogs, in which MSG was fed at dietary levels of up to 10% (Owen et al. 1978b). In these short-term (12 weeks) and long-term studies (104 weeks), it is clear that oral intake of MSG with diet, again the most probable route for people using MSG, does not have any adverse effects in any organ including the testes.
